# Mycobiota in Slovak wine grapes: A case study from the small Carpathians wine region

**DOI:** 10.1515/biol-2022-0676

**Published:** 2023-09-07

**Authors:** Soňa Felšöciová, Jozef Sabo, Natália Čmiková, Przemysław Łukasz Kowalczewski, Miroslava Kačániová

**Affiliations:** Institute of Biotechnology, Faculty of Biotechnology and Food Sciences, Slovak University of Agriculture in Nitra, Tr. A. Hlinku 2, 949 76 Nitra, Slovak Republic; Institute of Horticulture, Faculty of Horticulture and Landscape Engineering, Slovak University of Agriculture in Nitra, Tr. A. Hlinku 2, 949 76 Nitra, Slovak Republic; Department of Food Technology of Plant Origin, Poznań University of Life Sciences, 31 Wojska Polskiego St., 60-624 Poznań, Poland; Department of Bioenergetics and Food Analysis, Institute of Food Technology and Nutrition, University of Rzeszow, Zelwerowicza 4, 35-601, Rzeszow, Poland

**Keywords:** microscopic filamentous fungi, *Aspergillus* spp., *Penicillium* spp., mycotoxins, thin layer chromatography

## Abstract

The microbiological characteristics of the grapes are made up of a wide variety of microorganisms, including filamentous fungi. Their presence in grapes is traditionally associated with deterioration in quality. The health of the grapes is very important for obtaining quality wine. The objective of this study was to investigate the diversity of mycobiota on the surface and inside of different grapevine varieties at harvest time in the temperate climate of Slovakia and to identify potentially pathogenic isolates of *Aspergillus* and *Penicillium* producing selected mycotoxins. During the 2021 grape harvest, grapes were collected from the Small Carpathians wine region. Eleven grape samples were analyzed by the plating method and plating method with surface disinfection. Emphasis was placed on *Aspergillus* and *Penicillium* species because of their importance in mycotoxin production. Of the 605 fungal strains detected, 11 genera were identified in the exogenous mycobiota. The most common and abundant genera were *Alternaria* and *Botrytis.* In the genus *Aspergillus*, *A*. section *Nigri* is the most abundant, while in the genus *Penicillium*, *P. raistrickii* reached the highest frequency and abundance. Of the 379 strains detected and identified from the endogenous mycobiota, the most common genera were again *Alternaria* and *Botrytis* and the most abundant genus was *Botrytis. Penicillium* species were detected in 17% of all fungi found, with *P. raistrickii* dominating. The *A.* section *Nigri* reached only 4% of the relative density of all isolates. Potentially toxigenic *Aspergillus* and *Penicillium* species were tested for toxinogenity by thin layer chromatography. The most important mycotoxin-producing species found were *A*. section *Nigri* but without ochratoxin A production.

## 1 Introduction

Viticulture and winemaking in Slovakia are among the important traditional economic sectors. The area under vine in the Slovak Republic is 14,642 ha, of which 3,947 ha is in the Malokarpatska wine-growing region, 4,171 ha is in the South Slovak wine-growing region, 2,993 ha is in the Nitra wine-growing region, 1,713 ha is in the Central Slovak wine-growing region, 726 ha is in the East Slovak wine-growing region, and 1,092 ha is in the Tokaj wine-growing region [1].

Grapes, like other foods, can be contaminated by various fungi. Fungal diseases in the vineyard affect the quality of grapes by influencing the volatile profile and consequently affecting the aroma, flavor, and color of juice and wine. Molds synthesize numerous compounds e.g., *Aspergillus* and *Penicillium* produced 3-methylbutanol, 3-octanol, 1-octen-3-ol, 1-octanol, and 2-octen-1-ol along with a number of higher alcohols, aldehydes, and ketones [2]. Among these odors, 1-octen-3-ol is described as being “musty” and “mushroom.” Other compounds possessing “musty” or “moldy” sensory attributes are also synthesized. In addition to these sensory effects, the presence of filamentous fungi in grapes can result in mycotoxins. Mycotoxins are toxic secondary metabolites produced by molds that infect field crops, contaminate raw agricultural commodities as well as processed foods and feeds. Mycotoxins in food and feed can be fatal or cause severe illness at very low concentrations, often measured in parts per million or parts per billion. In addition to acute toxicosis due to high mycotoxin exposure, prolonged exposure to low and sublethal doses of mycotoxins leads to chronic toxicosis, which is cumulative damage to specific tissues, organs, or systems, sometimes accompanied by the development of cancer [3,4].

However, food and feed may be contaminated by several fungal species simultaneously or in rapid succession. Therefore, humans and animals are generally exposed to a cocktail of mycotoxins. Even very low doses of mycotoxins can synergistically impair the intestinal health, and the consequences of combined exposure to environmental and food contaminants are specific to the target organ [5]. The presence of filamentous fungi and toxin levels in grapes and wines vary depending on the grape variety, wine region, agricultural practices, weather conditions, harvest, storage, and winemaking practices [6].

Among the contaminating microorganisms, the mycotoxin-producing fungi *Aspergillus* and *Penicillium* are of particular concern [7,8]. Among mycotoxins in grapes, such as alternariol, aflatoxins (AFs), tenuazonic acid, fumonisin B_2_, ochratoxin A (OTA), patulin, citrinin, and others [9]. OTA is the main contaminant of grapes, juice, and wine [10,11]. This toxin has shown neurotoxic, genotoxic, carcinogenic, mutagenic, teratogenic, and immunosuppressive effects in animals [6]. *Aspergillus carbonarius* and *Aspergillus niger* are the main fungi responsible for the accumulation of OTA in grapes [10,11,12,13]. Although *A. carbonarius* is less frequently isolated than other black aspergilli, it is considered the most important producer of OTA because almost all isolates (if not all) produce the toxin, which occurs at high concentrations [14]. More often, two or more mycotoxins occur simultaneously [15]. OTA occurs more or less frequently together with minor *Aspergillus* and *Penicillium mycotoxins* such as citrinin and Penicillic acid, which also have nephrotoxic and carcinogenic effects, with AFs, or with the *Fusarium mycotoxin* fumonisin B_1_ [16]. Compared to other mycotoxins, patulin is considered less risky because the production of significant amounts of patulin is accompanied by visible rotting of fruit, so almost all of the toxins can be removed by eliminating rotten fruit through selection and sorting. Nevertheless, patulin concentrations in food are subject to regulatory control in some countries [3].

Sour rot and *Botrytis* infections are the most common causes of severe crop loss in grapes. Sour rot usually affects dense grapes just before harvest and is typically characterized by vinegar odor and brown berries [17]. *Botrytis* infections (also known as grey mold) often occur in vineyards exposed to cold and humid conditions during the ripening period and has a detrimental effect on the organoleptic properties [18]. Good agricultural practices and pre-harvest protection are very important to prevent pathogens from infecting crops and infected fruits from entering the food chain [19].

## 2 Materials and methods

### 2.1 Sampling of grape berries

In the 2021 vintage, the grape samples were collected from a small family winery in the village Vrbove (48°37′12″S 17°43′25″V), which is situated in Vrbovsky subregion, the part of the Small Carpathians wine region [20]. Three samples of red grape varieties (Alibernet, Blaufränkisch, and Cabernet Sauvignon) and eight samples of white grape varieties (Chardonnay, Irsai Oliver, Müller-Thurgau, Pálava, Pinot Blanc, Rheinriesling, Feteasca Regala, and Welschriesling) without visible signs of fungal infection were taken during the last ripening phase of the berries (harvest season), in September/October 2021. Ten plants were selected randomly along two diagonal rows of the vineyard and two bunches of grapes from each plant were harvested. Each of the grape sample was placed directly in a sterile plastic bag. The samples were taken to the laboratory and stored at 5°C until fungal analysis.

### 2.2 Mycological analysis of grape berry samples

We used two methods to cultivate and isolate the fungi: (a) direct plating without surface disinfection which corresponds to exogenous contamination and (b) direct plating with surface disinfection which corresponds to endogenous contamination. Fifty grape berries from each sample were randomly selected and then plated in Petri plates (140 mm diameter, 7–8 berries/dish) with Dichloran Rose Bengal Chloramphenicol Agar (Merck, Germany). Petri plates were incubated at 25°C for 7–10 days. Fifty additional grape berries were surface disinfected for 1 min in 1% NaClO according to the methods of Magnoli et al. [21] and rinsed three times with sterile distilled water (total volume 1 L) to remove incidental surface contamination, dried, plated in the same medium, and incubated for 5–7 days at 25 ± 1°C in the dark. Taxonomic identification of all isolates was performed by macroscopic and microscopic observation following the guidance of Pitt and Hocking [22]. *Aspergillus* strains were incubated on Czapek Yeast Agar (CYA; Merck, Germany) [23], Malt Extract Agar (MEA; Merck, Germany) [23], and Czapek Yeast Extract with 20% Sucrose (Merck, Germany) [22]. *Penicillium* strains were incubated on CYA, MEA, Creatine Sucrose Agar [23], and Yeast Extract Agar (YES; Merck, Germany) [23] incubated in 90 mm plastic Petri plates in the dark at 25 ± 1°C. After 7 days of incubation, the macroscopic and microscopic characteristics were observed according to the relevant mycological literature. Identification of *Aspergillus* strains was according to Klich [24], Pitt and Hocking [22], and identification of *Penicillium* strains was according to Pitt and Hocking [22], Samson et al. [25], and Frisvad and Samson [26].

### 2.3 Data analysis

The obtained results were evaluated and expressed in terms of relative density (RD) and isolation frequency (IF). The RD (%) is defined as the percentage of isolates of the species or genus present in the analyzed sample [27]. These values were calculated following González et al. [28] as follows:

(1) \[\text{RD}( \% )=(\text{ni}/\text{Ni})\times 100,]\]

where ni – number of isolates of a species or genus; and Ni – total number of isolated fungi.

IF (%) is defined as the percentage of samples in which the species or genus occurred at least once. These values were calculated following González et al. [28] as follows:

(2) \[\text{IF}( \% )=(\text{ns}/N)\times 100,]\]

where ns – number of samples with a species or genus, and N – total number of samples.

### 2.4 Evaluation of mycotoxigenic potential of Aspergillus and Penicillium isolates

To evaluate the toxigenic potential we used the agar plug method on thin layer chromatography (TLC) according to Samson et al. [25], modified by Labuda and Tančinová [29], in which potentially toxigenic species producing extracellular metabolites – OTA, AF B_1_, AF G_1_, citrinin, and patulin – were inoculated on YES agar and potentially toxigenic species producing intracellular metabolites – roquefortine C, penitrem A (PA), and cyclopiazonic acid (CPA) – were inoculated on CYA agar. From the colonies (after 14 days of incubation), some mycelial pieces of about 5 mm × 5 mm were cut and placed in an Eppendorf tube containing 500 μL chloroform:methanol in the ratio of 2:1 (Reachem, Slovak Republic). The contents of the tubes were shaken for 5 min with Vortex Genie^®^ 2 (MO BIO Laboratories, Inc., Carlsbad, CA, USA). Mycotoxins extracted from cultures of fungal isolates were determined by TLC technique on a precoated silica gel plate (Alugram^®^ SIL G, Macherey-Nagel, Germany). 30 μL of the liquid phase of the extracts was applied to the TLC plate together with 10 μL of standards (Sigma, Germany). The plate was placed in TEF solvent (toluene:ethyl acetate:formic acid; 5:4:1, toluene - Mikrochem, Slovak Republic; ethyl acetate and formic acid - Slavus, Slovak Republic). After elution, the plate was air dried. Mycotoxins were identified by comparison with appropriate reference standards for mycotoxins. Roquefortine C (RC) was visible as an orange spot after spraying with Ce(SO_4_)_2_ × 4H_2_O. PA was visible as a dark blue spot after spraying with 20% AlCl_3_ in 60% ethanol and heating at 130°C for 8 min. CPA was visible as a purple-tailed spot after spraying with Ehrlich reagent directly in daylight. Patulin was detected by spraying with 0.5% methylbenzothiazolone hydrochloride(Merck, Germany) in methanol and heating at 130°C for 8 min and was then visible as a yellow-orange spot. Directly under UV light with a wavelength of 365 nm, OTA was visualized as a blue-green spot, citrinin as a yellow-green-tailed spot, AF B_1_ as a blue spot, and AF G_1_ as a green-blue spot.

## 3 Results

### 3.1 Exogenous mycobiota from 11 grape varieties

**Figure 1:**
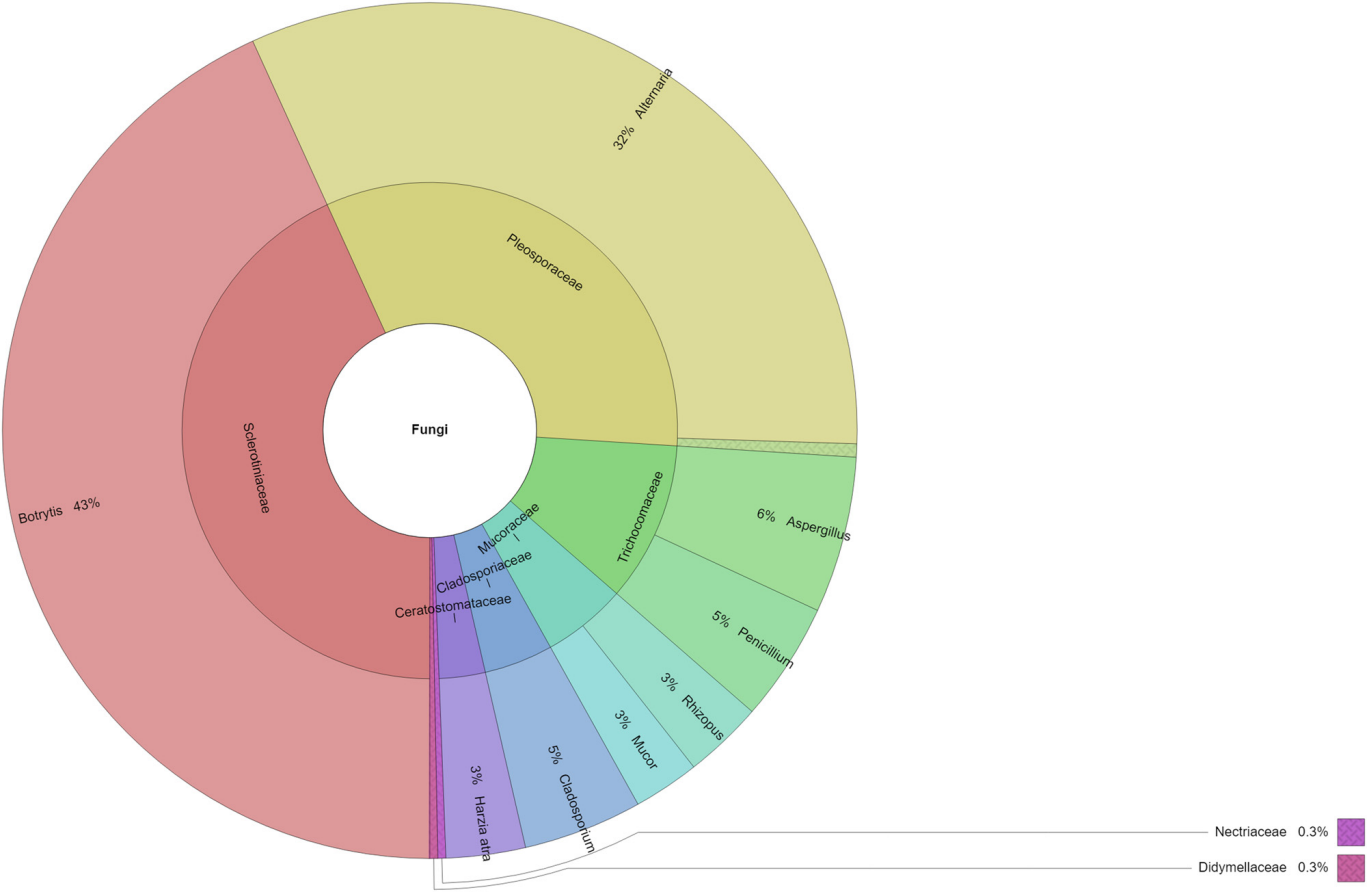
Krona chart: percentage of isolated microscopic fungi by the direct plating method.

The filamentous fungi of the surface mycobiota identified in eight white and three red grape varieties are shown in Table 1. A total of 605 strains belonging to 11 genera were identified. The highest number of isolates (from 82 to 103) belonging to 6 or 8 genera was found in Alibernet (1), Cabernet Sauvignon (3), and Pálava (7) varieties. A smaller number of isolates (38) were isolated from the white grape variety Chardonnay (4), but with a large number of genera (7). The fewest isolates (4) with a genus of microscopic filamentous fungi were isolated from the white grape variety Irsai Oliver (5). Almost all samples were colonized by the genera *Alternaria* and *Botrytis* (91% IF, each), followed by *Aspergillus* (82% IF), *Penicillium* (73% IF), *Cladosporium* and *Mucor* (54% IF, each), and *Rhizopus* (45% IF). The most abundant genus from exogenous mycobiota was *Botrytis* (43% RD, Figure 1), followed by *Alternaria* (32% RD) and *Aspergillus* (6% RD) of all fungi found. The other genera were detected in less than 5% of all isolates. Figure 1 shows the most isolated family of mycobiota by the direct plating method. The most isolated family was *Sclerotiniaceae* and genus *Botrytis* (43%), and *Pleosporaceae* and genus *Alternaria* (32%).

**Table 1:** Fungi identified in Slovak wine grapes, IF, and RD from exogenous mycobiota in 2021 by the direct plating method

Fungal taxa	1	2	3	4	5	6	7	8	9	10	11	No.	IF (%)	RD (%)
*Alternaria*	26	3	71	7	—	3	26	18	10	22	10	196	91	32
*Aspergillus*	1	11	4	3	—	—	1	1	5	3	5	34	82	6
*A. flavus*	—	—	—	—	—	—	—	1	—	—	—	1	9	3
*A.* section *Nigri*	1	11	4	3	—	—	1	—	5	3	5	33	73	97
*Botrytis*	51	30	19	2	—	14	34	12	32	36	32	262	91	43
*Cladosporium*	1	—	—	—	—	—	13	1	3	6	3	27	54	4
*Epicoccum*	—	2	1	—	—	—	—	—	—	—	—	3	18	<1
*Fusarium*	—	—	1	—	—	—	—	—	—	—	1	2	18	<1
*Harzia atra*	—	—	—	12	—	—	—	5	1	—	1	19	36	3
*Mucor*	1	—	—	4	4	—	3	2	1	—	—	15	54	2
*Penicillium*	14	1	—	6	—	1	1	2	1	—	1	27	73	4
*P. brevicompactum*	—	—	—	—	—	1	—	—	—	—	—	1	9	4
*P. crustosum*	—	—	—	1	—	—	—	2	—	—	—	3	18	11
*P. expansum*	—	—	—	—	—	—	1	—	—	—	—	1	9	4
*P. chrysogenum*	5	—	—	—	—	—	—	—	—	—	—	5	9	18
*P. polonicum*	—	—	—	1	—	—	—	—	—	—	—	1	9	4
*P. raistrickii*	9	—	—	—	—	—	—	—	1	—	1	11	27	41
*P. *spp.	—	1	—	4	—	—	—	—	—	—	—	5	18	18
*Phoma*	—	—	—	—	—	—	2	—	—	—	—	2	9	<1
*Rhizopus*	—	—	7	4	—	—	2	2	—	3	—	18	45	3
**Total**	94	47	103	38	4	18	82	43	53	70	53	605		

1–Alibernet, 2–Blaufränkisch, 3–Cabernet Sauvignon, 4–Chardonnay, 5–Irsai Oliver, 6–Müller-Thurgau, 7–Pálava, 8–Pinot Blanc, 9–Rheinriesling, 10–Feteasca Regala, 11–Welschriesling, No.–number of isolated micromycetes, IF–isolation frequency, RD–relative density.

### 3.2 Endogenous mycobiota from 11 grape varieties

**Figure 2:**
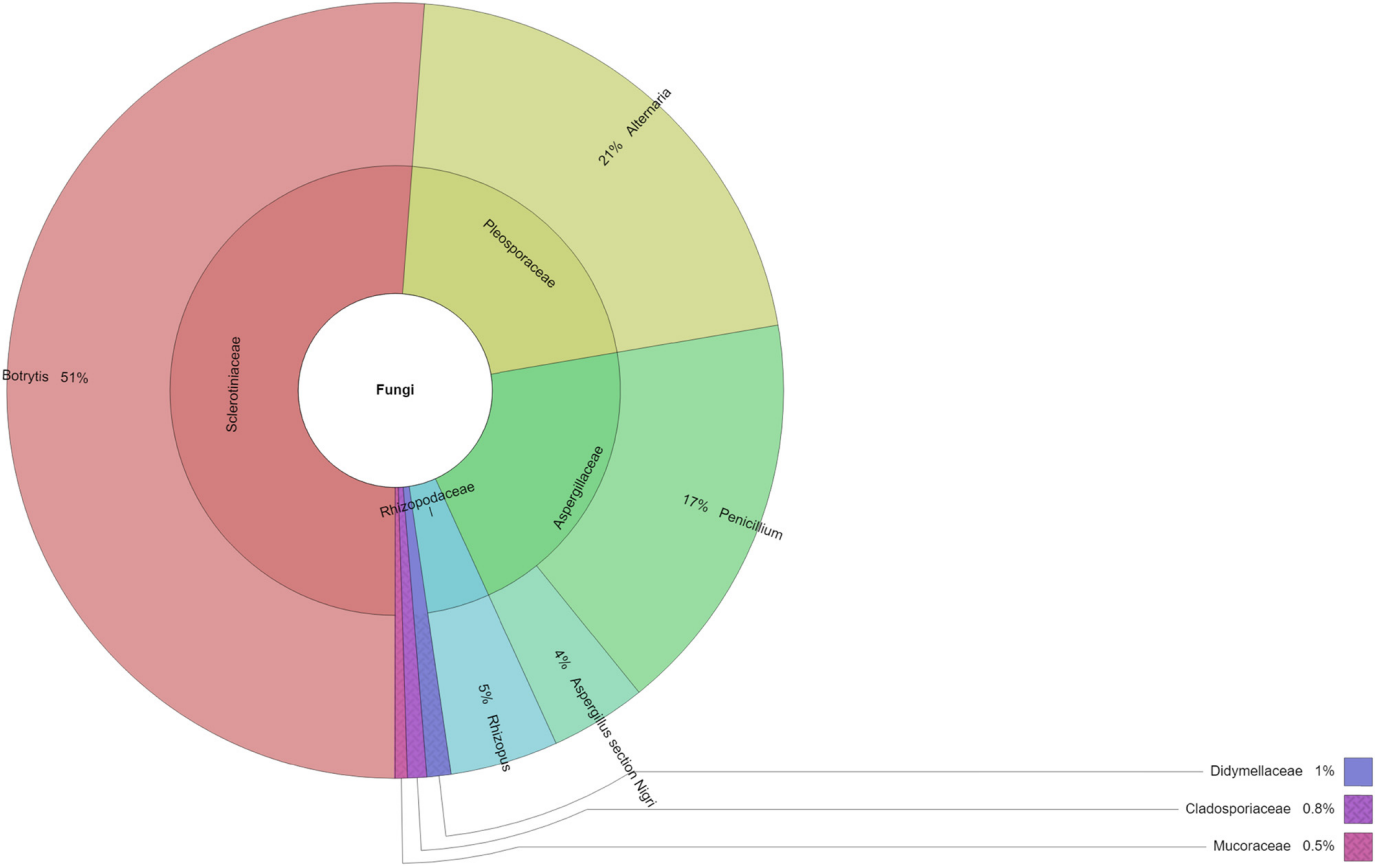
Krona chart: percentage of isolated microscopic fungi by the direct plating method with surface disinfection.

The filamentous fungi of the endogenous mycobiota identified in 11 grape varieties are shown in Table 2. A total of 379 strains belonging to 8 fungal genera were identified. The highest number of isolates (77 and 141) belonging to 5 and 6 genera was found in Alibernet (1) and Cabernet Sauvignon (3) cultivars. The highest number of genera (7) was found in the grape variety Rheinriesling (9). The lowest quantitative representation of micromycetes (4) with 4 genera was isolated from the white grape variety Pinot Blanc (8). Only 1 genus was found from the Irsai Oliver (5) grape variety. Almost all samples were colonized by the genera *Alternaria* and *Botrytis* (91% IF, each), followed by *Aspergillus*, *Penicillium* (54% IF, each), and *Rhizopus* (45% IF). *Botrytis* reached the highest number of isolates in most samples, except in two varieties of Chardonnay (4) and Feteasca Regala (10), where the genus *Alternaria* dominated, and *Alternaria* was not detected at all in 2 samples of Müller-Thurgau (6) and Pinot Blanc (8). The most abundant genus of endogenous mycobiota was *Botrytis* (51%, Figure 2), followed by *Alternaria* (21%) and *Penicillium* (17%) of all fungi found. The remaining genera were detected in less than 5% of all isolates. The most isolated family of mycobiota by the direct plating method with surface disinfection is shown in Figure 2. Similar to the direct plating method, the most isolated family was *Sclerotiniaceae* and genus *Botrytis* (51.2%), and *Pleosporaceae* and genus *Alternaria* (21.1%).

**Table 2:** Fungi identified in Slovak wine grapes, IF, and RD from endogenous mycobiota in 2021 by the direct plating method with surface disinfection

Fungal taxa	1	2	3	4	5	6	7	8	9	10	11	No.	IF (%)	RD (%)
*Alternaria*	16	2	24	11	—	2	2	1	4	14	4	80	91	21
*Aspergillus* section *Nigri*	1	2	1	—	—	1	—	1	9	—	—	15	54	4
*Botrytis*	47	5	65	2	10	—	27	—	18	8	12	194	91	51
*Cladosporium*	1	—	—	—	—	—	—	—	1	—	1	3	27	<1
*Epicoccum*	1	—	—	—	—	—	—	—	3	—	—	4	18	1
*Mucor*	—	—	—	—	—	—	—	—	1	1	—	2	18	<1
*Penicillium*	11	3	41	—	—	2	—	1	6	—	—	64	54	17
*P. brevicompactum*	—	—	—	—	—	1	—	—	—	—	—	1	9	1
*P. crustosum*	—	—	—	—	—	—	—	1	—	—	—	1	9	1
*P. chrysogenum*	4	—	11	—	—	—	—	—	4	—	—	19	27	30
*P. polonicum*	—	—	—	—	—	—	—	—	2	—	—	2	9	3
*P. raistrickii*	7	1	30	—	—	1	—	—	—	—	—	39	36	61
*P. *spp.	—	2	—	—	—	—	—	—	—	—	—	2	9	3
*Rhizopus*	—	—	10	4	—	—	1	1	—	1	—	17	45	4
Total	77	12	141	17	10	5	30	4	42	24	17	379	—	—

1–Alibernet, 2–Blaufränkisch, 3–Cabernet Sauvignon, 4–Chardonnay, 5–Irsai Oliver, 6–Müller-Thurgau, 7–Pálava, 8–Pinot Blanc, 9–Rheinriesling, 10–Feteasca Regala, 11–Welschriesling, No.–number of isolated micromycetes, IF–isolation frequency, RD–relative density.

### 3.3 Toxinogenity of *Aspergillus* and *Penicillium* isolates from selected grape varieties

Food safety is a priority for humanity. Mycotoxins pose a major threat to food safety [3]. The data in Table 3 show the toxicogenic profile of the 29 *Aspergillus* isolates and 52 *Penicillium* isolates representing *A. flavus*, *A.* section *Nigri*, *P. crustosum*, *P. expansum*, *P. chrysogenum*, and *P. raistrickii*. From the exogenous mycobiota of grapes, 34 isolates belonging to 2 *Aspergillus* and 4 *Penicillium* species were screened. All strains of *A.* section *Nigri* tested were negative for OTA, and one strain of *A. flavus* was negative for AF B_1_, G_1_, and CPA. Three strains of *P. crustosum* were positive for RC and PA, and one strain of *P. expansum* was positive for RC, citrinin, and patulin. A positive toxinogenity was detected for RC by *P. chrysogenum* (three out of five strains) and for griseofulvin by *P. raistrickii* (five out of six).

**Table 3:** Toxinogenity of selected *Aspergillus* and *Penicillium* strains isolated from exogenous and endogenous mycobiota of wine grapes

Species	OTA	Griseofulvin	Patulin	Citrinin	RC	PA	AFB_1_	AFG_1_	CPA
Toxinogenity of exogenous mycobiota									
*Aspergillus flavus*	—		—	—	—	—	0/1	0/1	0/1
*Aspergillus* section *Nigri*	0*/18**		—	—	—	—	—	—	—
*Penicillium crustosum*	—		—	—	3/3	3/3	—	—	—
*P. expansum*	—		1/1	1/1	1/1	—	—	—	—
*P. chrysogenum*					3/5				
*P. raistrickii*		5/6							
Toxinogenity of endogenous mycobiota									
*Aspergillus* section *Nigri*	0/10		—	—	—	—	—	—	—
*Penicillium chrysogenum*	—				12/14	—	—	—	—
*P. raistrickii*		18/23							

^*^ –number of isolates with the ability to produce mycotoxins, **–number of tested isolates, OTA–ochratoxin A, RC–roquefortin C, PA–penitrem A, AF–aflatoxin, CPA–cyclopiazonic acid.

Ten *Aspergillus* strains and 37 *Penicillium* strains isolated from the endogenous mycobiota of grapes were evaluated for their ability to produce mycotoxins. Negative toxinogenity for OTA by *Aspergillus* section *Nigri* was detected not only from exogenous but also from endogenous mycobiota. The ability to produce RC by *P. chrysogenum* in *in vitro* conditions was detected in 12 out of 14 strains and griseofulvin by *P. raistrickii* in 18 out of 23 strains.

## 4 Discussion

In previous studies [30,31], *Alternaria* was also the most frequently occurring genus with the highest RD in Slovak grapes from the Small Carpathians wine region, the South Slovak wine region [32], and the Central Slovak wine region [33]. *Botrytis* was a less frequently occurring fungal genus with a low RD in grape samples [21,32,33]. The ascomycete *Botrytis cinerea* is considered to be one of the most destructive fungi in viticulture in cool climates, causing *Botrytis* bunch rot or grape grey mold and dramatically changing the physiochemical state of grape berries [34] or noble rot in “botrytized” grapes that produce the greatest white wines [35]. *Aspergillus, Alternaria*, *Botrytis*, *Penicillium*, and *Cladosporium* were five of the most frequent genera in all four winemaking regions in Portugal [8]. In our study, contamination by *Aspergillus* was higher than that by *Penicillium* in most samples, except for Alibernet, Chardonnay, and Pinot Blanc, in which *Penicillium* was more represented. However, the diversity of *Penicillium* species was higher than that of *Aspergillus*. Kizis et al. [36] studied the colonization of grapes by filamentous fungi from growing areas in Greece. *Aspergillus* was the most frequently isolated genus in all regions except Macedonia, where the genus *Alternaria* was most common. EL Khoury et al. [37] found similar results in Lebanese vineyards, where 95.5% of the isolates belonged to the genus *Aspergillus*, while only 4.5% belonged to the genus *Penicillium*. The *Aspergillus* species found in our grapes from the Vrbovsky subregion were *A. flavus* and *A.* section *Nigri*. The species of section *Nigri* accounted for 97% of the *Aspergillus* isolates. These results are consistent with the reports by Battilani et al. [38], who found that *A.* section *Nigri* was the most commonly isolated species in hot and dry areas. Among them, *A. carbonarius* and *A. niger* were the most abundant species in grapes [7]. Freire et al. [6] found similar results in Brazilian vineyards. Species of the *A.* section *Nigri* were predominant in the mycobiota of grapes from the Small Carpathians area in Slovakia during 2011–2013 harvest [30,32]. Certainly, *Aspergillus* species is present worldwide, in all wine grape products, and under all environmental conditions, most frequently in warmer regions and on heat-producing substrates [39]. The toxigenic species *A. clavatus* and *A. flavus* were the other main species recorded with a high IF. The identified species of the genus *Penicillium* were P*. raistrickii*, *P. chrysogenum*, *P. crustosum*, *P. brevicompactum*, *P. expansum*, and *P. polonicum*. Most cultivars were colonized by *P. raistrickii*, which dominated in 27% of the samples examined and accounted for 41% of *Penicillium* isolates, especially in the Alibernet cultivar. *Penicillium raistrickii* is a soil fungus and has a widespread though sparse distribution in food [22]. These results differ from those in Brazilian vineyards, where the most abundant *Penicillium* species were *P. sclerotiorum*, *P. citrinum*, *P. glabrum*, *P. decumbens*, *P. implicatum*, and *P. solitum* [6]. *Penicillium brevicompactum*, *P. thomii*, and *P. glabrum* were found in Portuguese vineyards [8]. *Penicillium chrysogenum* was the most frequently isolated species in Argentina [21] and Slovakia [40]. In France and Portugal, *P. brevicompactum* was the species with the highest occurrence [41]. However, other studies identified *P. expansum* as the most common species in Portuguese [42], French [43], and Slovakian [30,32,40] vineyards. These species were reported in our study, too, but in the lowest frequency of occurrence.

In our study, almost all samples were colonized by the genera *Alternaria* and *Botrytis* in the endogenous mycobiota of grapes. *Alternaria* was the predominant genus in the endogenous mycobiota of grapes, and also in the Small Carpathians region [34], in the Central Slovak wine region [33], and in the Eastern Slovak wine region [40]. *Penicillium* is a common component of grapes and soils in temperate regions [8]. From the endogenous mycobiota, 64 strains of 5 *Penicillium* species were isolated, namely *P. brevicompactum*, *P. crustosum*, *P. chrysogenum*, *P. polonicum*, and *P. raistrickii*. Six samples were colonized by *Penicillium* species. The grape variety Cabernet Sauvignon was the most colonized by the species *P. raistrickii*. On the other hand, *Penicillium* was less frequently detected in grapes from the Small Carpathians area in 2018 with these four *Penicillium* species: *P. citrinum*, *P. griseofulvum*, *P. hordei*, and *P. chrysogenum* [31]. In our study, *Aspergillus* was as frequently occurring genus as *Penicillium*, but its RD was lower. All 15 isolates were identified as *A.* section *Nigri*. A similar conclusion was also reached by other authors [30,32,40,44,45,46].

Production of OTA was not confirmed in grapes from some Slovak wine-growing zones such as the Central Slovak wine region [34], the Eastern Slovak wine region [41], the Tokaj wine region [30], or the Small Carpathians wine region [40]. These results differ from other reports demonstrating OTA contamination in *Vitis vinifera* grapes [12,38,39,47,48,49,50,51,52]. Three strains of *P. crustosum* from our samples were positive for RC and PA, and one strain of *P. expansum* was positive for RC, citrinin, and patulin. *Penicillium* was a common component of grapes from the Small Carpathians area from 2011 to 2013 [40] where positive toxinogenity was detected in *P. crustosum* on PA and RC (14/14, 100%). *Penicillium expansum* produced RC (5 out of 5), patulin (3 out of 5), and citrinin (2 out of 5). Patulin is considered less risky because production of significant amounts of patulin is associated with visible rotting of fruit, so almost all of the toxin can be removed by rejecting rotten fruit through selection and sorting. Nevertheless, patulin concentrations in food are subject to regulatory control in some countries [3].

## 5 Conclusion

The present study provides a detailed description of the filamentous fungi found on the berry surface and inside of Slovak grapes collected from the wine region largest in size and the most important over the centuries, the Small Carpathians wine region in 2021. The results show *Botrytis* and *Alternaria* as the most frequent and abundant genera that can affect the sensory profile of must and wine. OTA, griseofulvin, patulin, citrinin, RC, PA, AF B_1_, AF G_1_, and CPA production were tested by TLC method in *in vitro* condition. Although several grape samples analyzed were contaminated with *A*. section *Nigri*, the abundance of *Aspergillus* was minimal and none of the isolates produced OTA. This is of crucial importance for understanding the fungal hazards for grapes and subsequently for wine and for the knowledge of field ecology of the fungal species. We recommend regular monitoring of mycological colonization of grapes, especially during harvest, with a focus on toxinogenic genera. Attention should also be focused on research aimed at mitigating mycotoxins in grapes or wine.
